# *MiR-101* Is Involved in Human Breast Carcinogenesis by Targeting *Stathmin1*

**DOI:** 10.1371/journal.pone.0046173

**Published:** 2012-10-11

**Authors:** Rui Wang, Hong-Bin Wang, Chan Juan Hao, Yi Cui, Xiao-Chen Han, Yi Hu, Fei-Feng Li, Hong-Fei Xia, Xu Ma

**Affiliations:** 1 Reproductive and Genetic Center of National Research Institute for Family Planning, Beijing, China; 2 Graduate School, Peking Union Medical College, Beijing, China; 3 The Third Affiliated Hospital of Harbin Medical University, Harbin, China; 4 Tangshan People's Hospital, Tangshan, China; King Faisal Specialist Hospital & Research Center, Saudi Arabia

## Abstract

**Background:**

*MicroRNA-101 (miR-101*) expression is negatively associated with tumor growth and blood vessel formation in several solid epithelial cancers. However, the role of *miR-101* in human breast cancer remains elusive.

**Results:**

*MiR-101* was significantly decreased in different subtypes of human breast cancer tissues compared with that in adjacent normal breast tissues (*P<*0.01). Up-regulation of *miR-101* inhibited cell proliferation, migration and invasion, and promoted cell apoptosis in ER alpha-positive and ER alpha-negative breast cancer cells and normal breast cells. Down-regulation of *miR-101* displayed opposite effects on cell growth and metastasis. Further investigation revealed a significant inverse correlation between the expression of *miR-101* and *Stathmin1* (*Stmn1*), and *miR-101* could bind to the 3′-untranslated region (UTR) of *Stmn1* to inhibit *Stmn1* translation. The inhibition of cell growth and metastasis induced by up-regulation of *miR-101* was partially restored by overexpresson of *Stmn1*. Knockdown of *Stmn1* attenuates the down-regulation of *miR-101-*mediated enhancement of cell growth and metastasis. More importantly, *in vivo* analysis found that *Stmn1* mRNA and protein level in different subtypes of human breast cancer tissues, contrary to the down-regulation of *miR-101*, were significantly elevated.

**Conclusions:**

This study demonstrates that down-regulation of *miR-101* in different subtypes of human breast cancer tissues is linked to the increase of cellular proliferation and invasiveness via targeting *Stmn1*, which highlights novel regulatory mechanism in breast cancer and may provide valuable clues for the future clinical diagnosis of breast cancer.

## Introduction

MicroRNAs (miRNAs) are endogenous 19–25 nucleotide non-coding single-stranded RNAs that regulate gene expression by blocking the translation or decreasing the stability of mRNAs [1]. In fact, almost one-third of the protein-coding genes are susceptible to miRNA regulation [2]. Therefore, many miRNAs seem to play pivotal roles in many biological processes, including cellular proliferation, differentiation and apoptosis [3]. In recent years, accumulating evidence indicates that dysregulation of miRNAs is associated with the initiation and progression of cancer. Several studies have shown that unique miRNA expression profiles are present in lots of cancers, such as breast, lung, oesophageal, prostate and pancreatic cancer, gastric cancer and colon cancer, suggesting that miRNAs may act as oncogenes or cancer suppressors [4].

Breast cancer is the most commonly malignancies in women. Since the year of 2005, miRNAs was firstly reported to have a close relationship with breast cancer, many miRNAs were found to be associated with breast cancer. For example, *miR-200* enhances mouse breast cancer cell colonization to form distant metastases [5]. *MiR-34a* expression has an effect for lower risk of metastasis and associates with expression patterns predicting clinical outcome in breast cancer [6]. MiR-203/SNAI1 feedback loop regulates epithelial to mesenchymal transition in human breast cancer cells [7]. Circulating ectopic *miR-125b* expression increases the resistance to anticancer drug *in vitro* in breast cancer cells [8]. Downregulation of *miR-92a* is associated with aggressive breast cancer features and increases tumor macrophage infiltration [9]. Expression of *miR-210* is linked to tumor proliferation and appears to be a strong potential biomarker of clinical outcome in breast cancer [10]. These reports demonstrate that miRNAs play an important role in the breast cancer progression.

MiR-101 belongs to a family of miRNAs that are involved in a series of cellular activities, e.g. cell proliferation, invasion, angiogenesis [11], [12]. MiR-101-1 has been found in the genomic fragile regions that are associated with abnormal deletion or amplification in cancer [13]. In recent years, several published studies have shown that *miR-101* is obviously down-regulated in different types of cancer, e.g. glioblastoma, non-small cell lung cancer or human colon cancer. The diminution of *miR-101* promoted the proliferation, migration and angiogenesis of cancer cells partly by targeting the *Enhancer of Zeste homology 2* gene or *cyclooxygenase-2* [11], [14]. Ectopic expression of *miR-101* could inhibit proliferation and invasion of gastric cancer cells [15], and sensitize the tumor cells to radiation *in vitro* and *in vivo* [16]. In addition, overexpression of *miR-101* can also inhibit normal mammary gland epithelial cell proliferation that influences the differentiation state of the mammary gland via altering *cyclooxygenase-2* expression [17]. Most importantly, *miR-101* is differentially expressed between breast tumors and normal breast tissues [18]. However, limited knowledge is available about the pathophysiological significance of *miR-101* in breast cancer.

In the present study, we defined the expression profile of *miR-101* and its target gene, *Stathmin1* (*Stmn1*), in breast cancer tissue and adjacent normal breast tissue, and studied the pathophysiological significance of *miR-101* in breast cancer using *in vitro* cell model. Our results demonstrated that *miR-101* expression was significantly down-regulated in different subtypes of human breast cancer tissues. An obvious inverse correlation between the expression of *miR-101* and *Stmn1* was demonstrated. The expression of *Stmn1* was significantly increased as *miR-101* decreased in human breast cancer tissues. The decrease of *miR-101* promoted cell proliferation, migration and invasion, and inhibited cell apoptosis by targeting *Stmn1*. These results suggest that *miR-101* may act as a tumor suppressor and can be a novel candidate gene for the diagnosis and therapy to different subtypes of breast cancer.

## Results

### Down-Regulation of *miR-101* Expression in Human Breast Cancer Tissues

**Figure 1:**
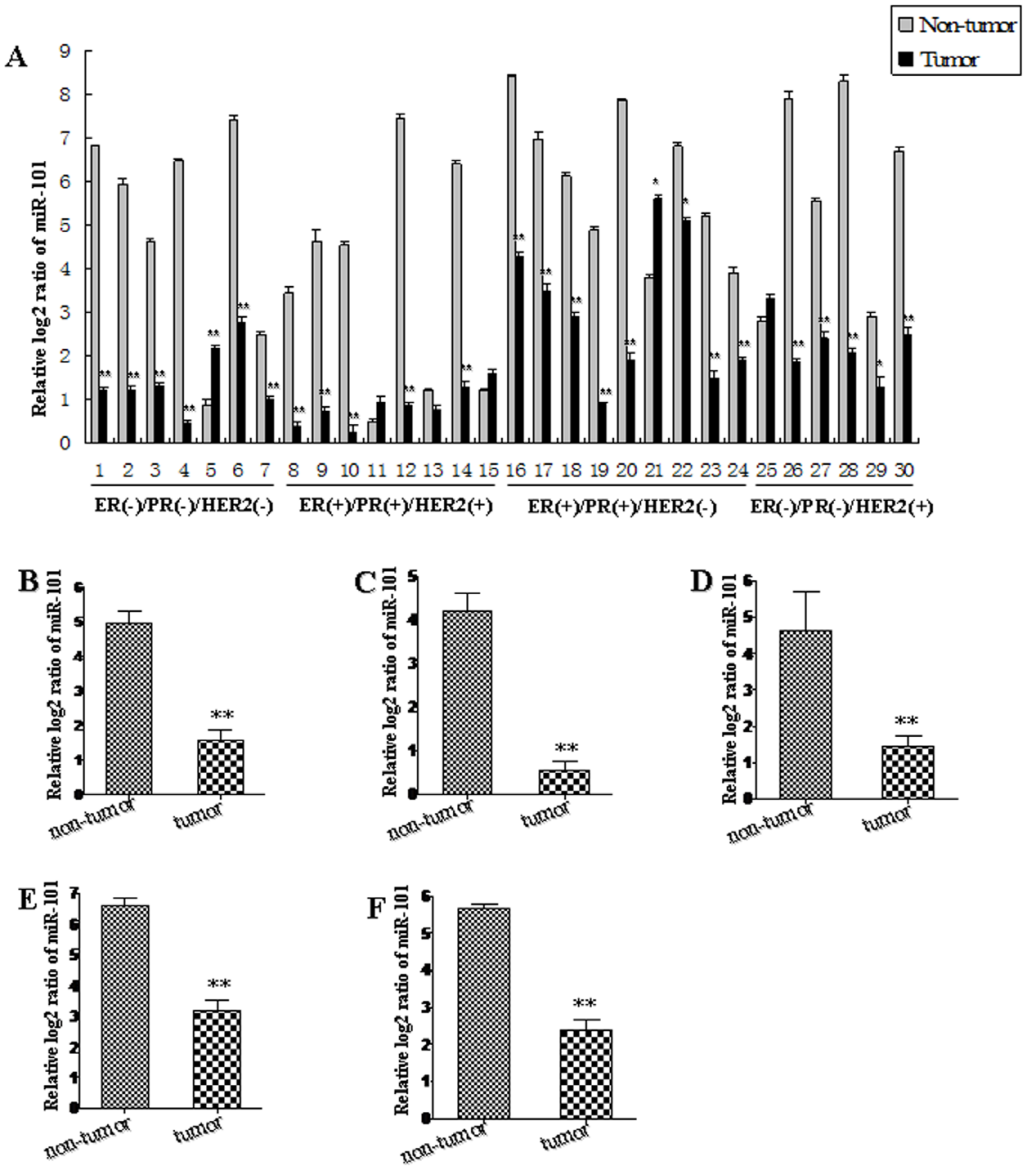
Down-regulation of *miR-101* in breast cancer tissues. We pooled two patients' cancer tissues as a sample. The expression level of *miR-101* in individual sample was detected by TaqMan miRNA RT-Real Time PCR (A). Statistical analyses were performed to analyze the overall trend of *miR-101* in all human breast cancer tissues (B), ER/PR−,HER2− (C), ER/PR+, HER2+ (D), ER/PR+, HER2− (E) and ER/PR−, HER2+ (F) breast cancer tissues. Relative expression of *miR-101* in breast cancer tissues and adjacent normal breast tissues was compared to U6 in corresponding tissue sample, i.e. the relative level of *miR-101* was normalized to U6. U6 serves as an internal reference among different samples and helps normalize for experimental error. The *y*-axis displays the relative log2 ratio of *miR-101* normalized by *U6*. *P<0.05, **P<0.01.

In order to explore the role of *miR-101* in breast carcinogenesis, the expression patterns of *miR-101* in 60 pairs of human breast cancer tissues and adjacent normal breast tissues were analyzed using qRT-PCR (Table 1; Figure 1). We pooled two patients' cancer tissues as a sample (30 samples for 60 pairs). *MiR-101* level was significantly decreased in 80% (24/30 samples) of breast cancer tissues (Figure 1A). In general, the expression of *miR-101* was significantly decreased in all human breast cancer tissues compared with that in adjacent normal breast tissues (*P<*0.01; Figure 1B). The breast cancer tissues were grouped into four subtypes according to the expression of estrogen receptor (ER), progesterone receptor (PR) and human epidermal growth factor receptor 2 (HER2), included ER/PR and HER2 negative (ER/PR-,HER2−; triple negative), ER/PR and HER2 positive (ER/PR+, HER2+), ER/PR positive and HER2 negative (ER/PR+, HER2−) and ER/PR negative and HER2 positive (ER/PR−, HER2+) breast cancer, respectively. The results from the analysis of the trend of *miR-101* expression in four different subtypes of breast cancer tissues were consistent with that in all breast cancer tissues. *MiR-101* expression was reduced in breast cancer samples of 85.71% (6/7 samples) of triple negative, 62.50% (5/8 samples) of ER/PR+, HER2+, 88.89% (8/9 samples) ER/PR+, HER2− and 83.33% (5/6 samples) of ER/PR−, HER2+, when compared with that in adjacent normal breast tissues, respectively (Figure 1A). The overall expression of *miR-101* was significantly decreased in four subtypes of human breast cancer tissues (*P<*0.01; Figure 1C–F). These results imply that *miR-101* is sensitive to different types of breast cancer, i.e. ER positive, HER2 positive and triple negative.

**Table 1:** Clinicopathologic characteristics of patients and breast tumors.

Clinical parameters	Cases (n = 60)	Control (n = 60)
ER PR HER2		
ER(+)/PR(+)/HER2(+)	14 (60)	14 (60)
ER(−)/PR(−)/HER2(−)	15 (60)	15 (60)
ER(+)/PR(+)/HER2(−)	19 (60)	19 (60)
ER(−)/PR(−)/HER2(+)	12 (60)	12 (60)
Tumor size (cm)		
≤2	26 (60)	26 (60)
>2	34 (60)	34 (60)
No. of positive lumph nodes		
0	29 (60)	29 (60)
1–3	13 (60)	13 (60)
>3	18 (60)	18 (60)
TNM stage		
I	9 (60)	9 (60)
II	39 (60)	39 (60)
II–III	5 (60)	5 (60)
III	7 (60)	7 (60)

**Figure 2:**
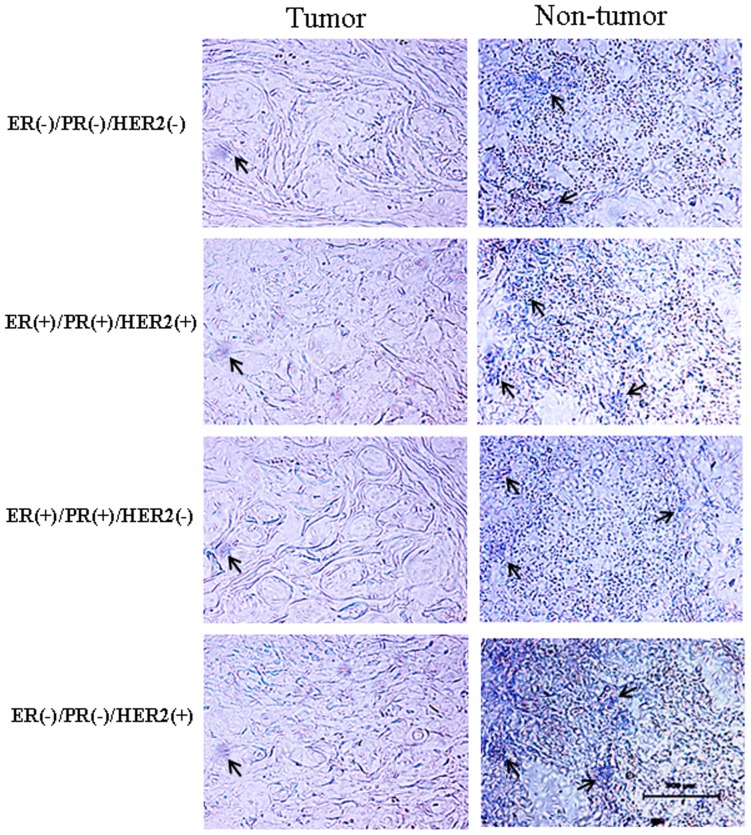
The distribution of *miR-101* in breast tissues. The location of *miR-101* in breast cancer tissues and adjacent normal breast tissues was subjected to *in situ* hybridization using DIG-labeled LNA probes specific to *miR-101.* The sections were hybridized with DIG-labeled LNA scrambled miRNA probe as a negative control (Figure S1A). The stain was developed with BCIP/NBT. Black arrows indicate hybridization signal. The scale bar indicated a distance of 500 µm.

The distribution of *miR-101* in human breast cancer tissues and adjacent normal breast tissue was determined by in situ hybridization (Figure 2). *MiR-101* was weakly detected in breast cancer tissues. Strong signals of *miR-101* were found in normal breast tissues. *MiR-101* was mainly localized in breast epithelial and stromal cells. No signal was detected in sections of normal breast tissues that were hybridized with the DIG-labeled LNA-scrambled probe as negative control (Figure S1A).

### *Mir-101* Regulates the Breast Cell Viability *in vitro*

**Figure 3:**
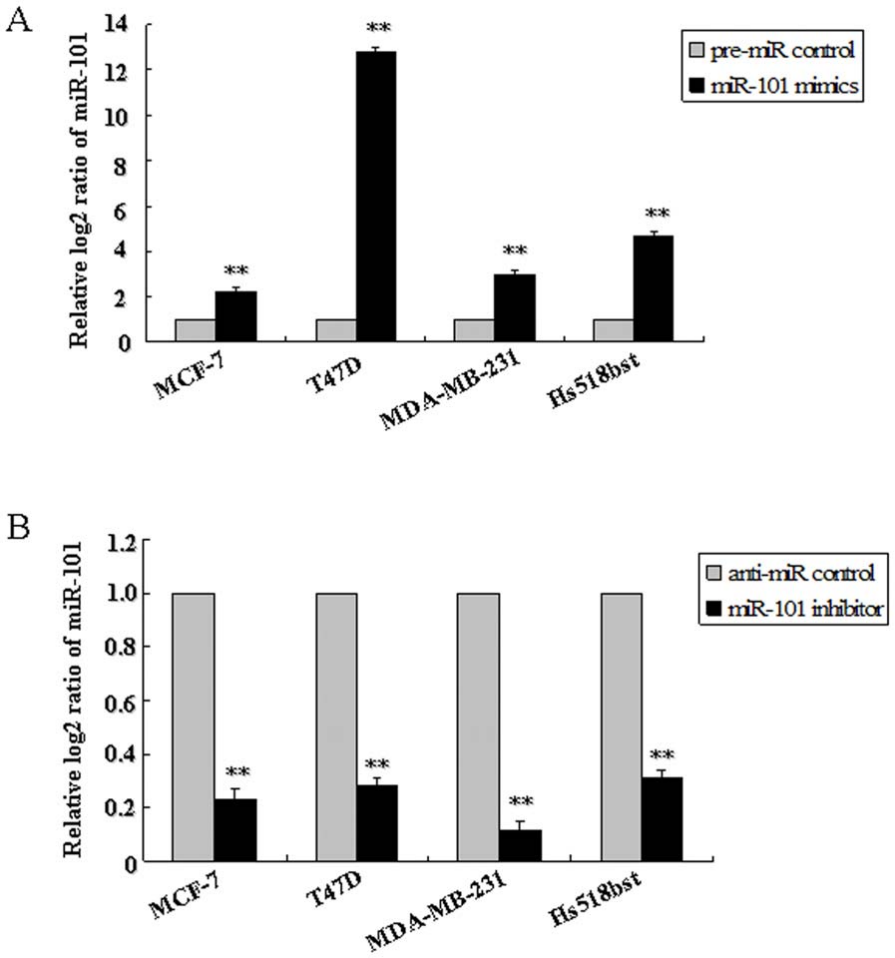
The effect of *miR-101* constructs on the expression level of *miR-101*. MCF-7, T47D, MDA-MB-231 and Hs518bst were respectively transfected with pre-miR control, *miR-101* mimics (A), anti-miR control or *miR-101* inhibitor (B). The *miR-101* level was detected by TaqMan miRNA RT-Real Time PCR. U6 serves as an internal reference. The expression of *miR-101* in control group was set to 1. The *y*-axis displays the relative log2 ratio of *miR-101* normalized by *U6*. **P<0.01.

To assess the possible function of *miR-101* in the pathological process of breast cells, the effect of *miR-101* on the growth of breast cells was detected using *in vitro* cell lines model. Firstly, the endogenous *miR-101* level in T47D (ER alpha-positive), MCF-7 (ER alpha-positive), MDA-MB-231 (ER alpha-negative) and Hs518bst (non-malignant mammary gland epithelial cell) was detected by qRT-PCR. Results showed that the *miR-101* level was higher in Hs518bst than MCF-7 (*P<*0.01) and MDA-MB-231 (*P<*0.01), similar with T47D (Figure S2). Hereafter, the effect of *miR-101* constructs on the expression level of *miR-101* in MCF-7, T47D, MDA-MB-231 and Hs518bst was detected by qRT-PCR. These cells were transfected with *miR-101* mimics, pre-miR control, *miR-101* inhibitor or anti-miR control, respectively. The results showed that *miR-101* expression level was markedly enhanced in cells transfected with *miR-101* mimics compared with pre-miR control (*P<*0.01), and remarkably decreased in cells transfected with *miR-101* inhibitor compared with pre-miR control (*P<*0.01) in MCF-7, T47D, MDA-MB-231 and Hs518bst cells (Figure 3).

**Figure 4:**
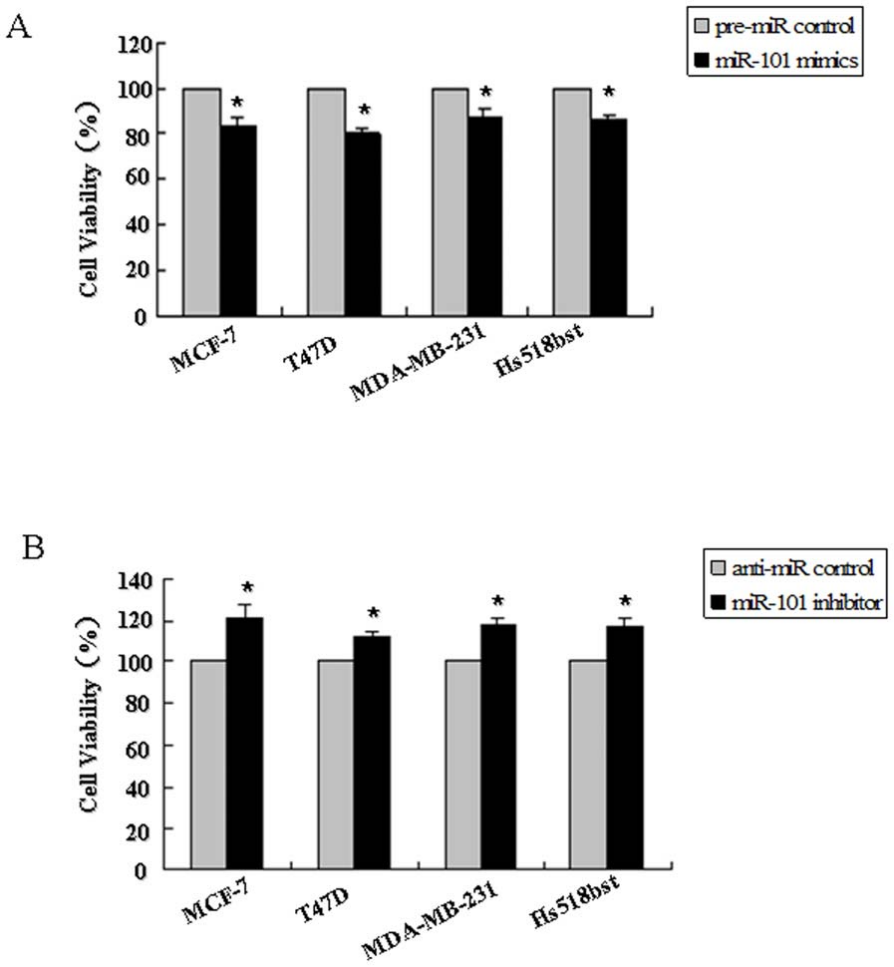
The effects of *miR-101* on proliferation of breast cells. MCF-7, T47D, MDA-MB-231 and Hs518bst cells were transfected with the pre-miR control, *miR-101* mimics (A), anti-miR control or *miR-101* inhibitor (B), respectively. After 48 h of transfection, cell proliferation was determined by MTT assay. All experiments were performed at least three times and calculated cell proliferation as stimulation index (SI) (ratio of A570 nm with *miR-101* mimics or inhibitor vs pre-miR or anti-miR control). *P<0.05.

The cell growth curve in these breast cell lines transfected by *miR-101* mimics or inhibitor from 12 hour to 72 hour was detected in order to evaluate the growth characteristics of cell lines (Figure S3). After 48 h of transfection, the cells were still in the exponential phase. Then the cell proliferation and viability in *miR-101* mimics or inhibitor-treated cells were determined by MTT assay 48 h after transfection. As shown in Figure 4, the relative proliferation rates in MCF-7, T47D, MDA-MB-231 and Hs518bst cells transfected with *miR-101* mimics were decreased about 15.95%, 19.75%, 12.09% and 13.7%, respectively, as compared with pre-miR control (*P<*0.05). The relative proliferation rates in MCF-7, T47D, MDA-MB-231 and Hs518bst cells transfected with *miR-101* inhibitor was increased about 21.82%, 12.56%, 18.20% and 17.5%, respectively, as compared with anti-miR control (*P<*0.05). These results show that re-expression of *miR-101* significantly suppresses breast cell viability, while down-regulation of *miR-101* evidently promotes breast cell proliferation in not only ER alpha-positive but also ER alpha-negative breast cancer cells and non-malignant mammary gland epithelial cells.

### *Mir-101* Affects Breast Cell Apoptosis *in vitro*

**Figure 5:**
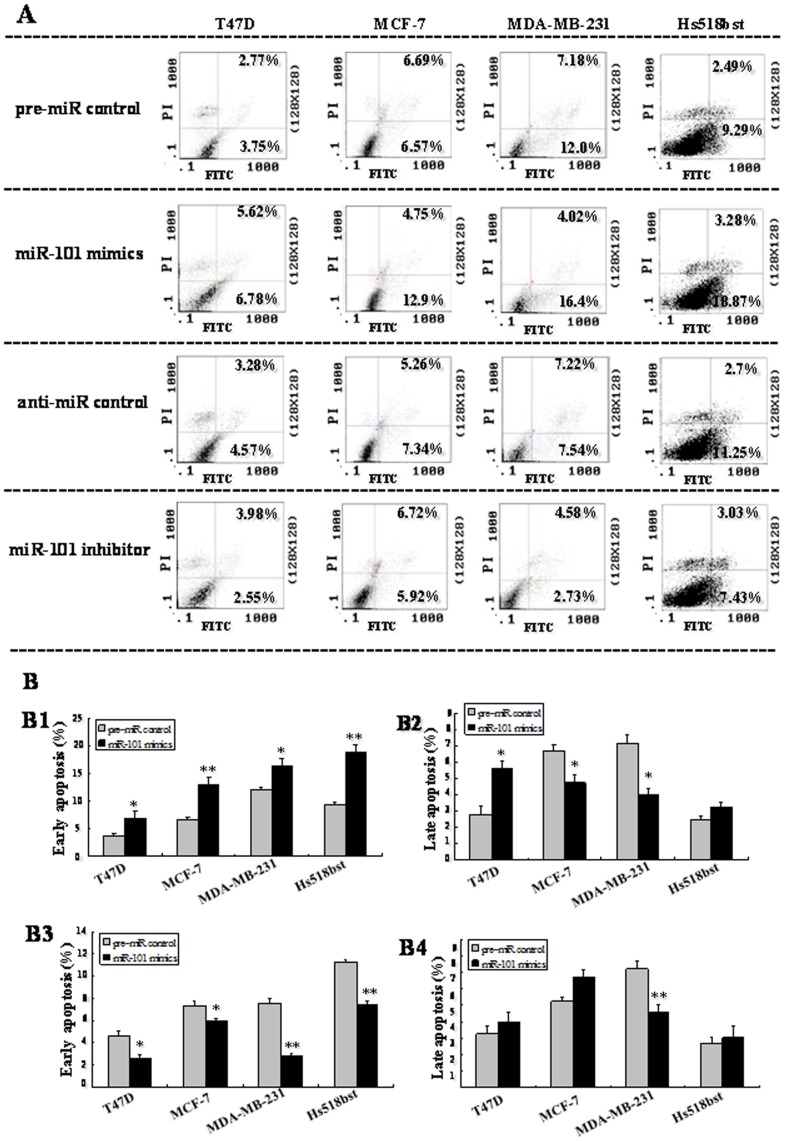
Apoptosis in breast cells were detected by flow cytometry. T47D, MDA-MB-231, MCF-7 and Hs518bst cells were transfected with the pre-miR control , *miR-101* mimics, anti-miR control, or *miR-101* inhibitor, respectively, for 48 h. Then single cell suspension was prepared and stained with annexin V/PI and subjected to flow cytometry analysis (A). Lower left quadrant, viable cells (annexin V-FITC and PI negative); lower right quadrant, early apoptotic cells (annexin V-FITC positive and PI negative); upper right quadrant, late apoptosis/necrosis cells (annexin V-FITC and PI positive). The percentage of early and late apoptotic cells (representatives of three separate experiments) is shown in the lower right and upper right panels, respectively. The average percentage of apoptosis cells was analyzed in cells transfected by *miR-101* mimics and inhibitor at early and late stages (B). The histograms respectively represent the average percentage of apoptosis cells in cells treated by *miR-101* mimics at early (B1), late stage (B2), and *miR-101* inhibitor at early (B3) and late stages (B4). The experiment was repeated at least three times.

To further explore the role of *miR-101* in controlling the growth of breast cells, apoptosis in four breast cells were determined by flow cytometry (Figure 5). In this assay, cells were stained with Annexin V-conjugated FITC (which was used to detect apoptotic cells with phosphatidylserine externalization) and PI. The cells in the lower right quadrant of the square chart (annexin V-FITC positive) represent the number of early apoptotic cells and in the upper right quadrant (annexin V-FITC/PI positive) represent late apoptotic cells.

We found that transfection with *miR-101* mimics increased the number of early apoptotic cells compared to pre-miR control in T47D (6.78% vs 3.75%; *P<*0.05), MCF-7 (12.9% vs 6.57%; *P<*0.01), MDA-MB-231 (16.4% vs 12.0%; *P<*0.05) and Hs518bst cells (18.87% vs 9.29%; *P<*0.01). The effect of *miR-101* on the number of late apoptotic cells varied with the breast cell lines. After transfection with *miR-101* mimics, the number of late apoptotic cells was increased in T47D (5.62% vs 2.77%; *P<*0.05) and decreased in MCF-7 (4.75% vs 6.69%; *P<*0.05) and MDA-MB-231 (4.02% vs 7.18%; *P<*0.05) compared to pre-miR control, and there was no significant difference in Hs518bst cells. When cells were transfected by *miR-101* inhibitor, the number of early apoptotic cells was decreased compared to anti-miR control in T47D (2.55% vs 4.57%; *P<*0.05), MCF-7 (5.92% vs 7.34%; *P<*0.05), MDA-MB-231 (2.73% vs 7.54%; *P<*0.01) and Hs518bst cells (7.43% vs 11.25%; *P<*0.01). The cells undergoing apoptosis at late stage varied with the breast cell lines after *miR-101* inhibitor treatment. The number of late apoptotic cells was reduced by the transfection with *miR-101* inhibitor in MDA-MB-231 (4.58% vs 7.22%; *P<*0.01) compared to anti-miR control, and there was no significant difference in T47D, MCF-7 and Hs518bst cells. These results show that up-regulation of *miR-101* obviously promotes cell apoptosis and down-regulation of *miR-101* significantly suppresses cell apoptosis at early stage.

### *Mir-101* Modulates Migration and Invasion Capacity of Breast Cells *in vitro*

**Figure 6:**
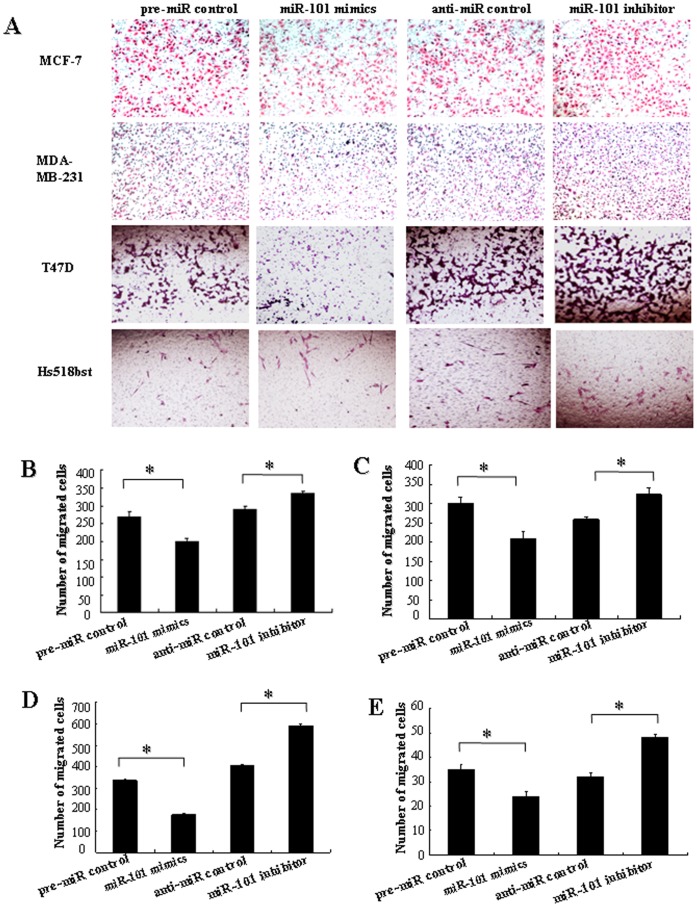
The effects of *miR-101* on cell migration of breast cells. T47D, MDA-MB-231, MCF-7 and Hs518bst cells were transfected with pre-miR control, *miR-101* mimics, anti-miR control or *miR-101* inhibitor, respectively. Cells were harvested 48 h after transfection and recounted to 0.5×10^6^ cells/ml in every group to seed Transwells for cell migration assay. At time of harvest, the cells on top of the membranes were removed, and the cells on the bottoms of the membranes were stained with haematoxylin and eosin. The cell migration was quantified by counting the amount of cells passing through the membrane from five different fields per sample at 100× selected in a random manner after 17 h of incubation. A show the representative photomicrographs of cells passing through the membrane at ×100 original magnification. Histogram B-E represents the number of migrated cells in MCF-7 MDA-MB-231, T47D and Hs518bst cells, respectively. Data are expressed as the mean numbers of independent triplicate experiments. *P<0.05.

**Figure 7:**
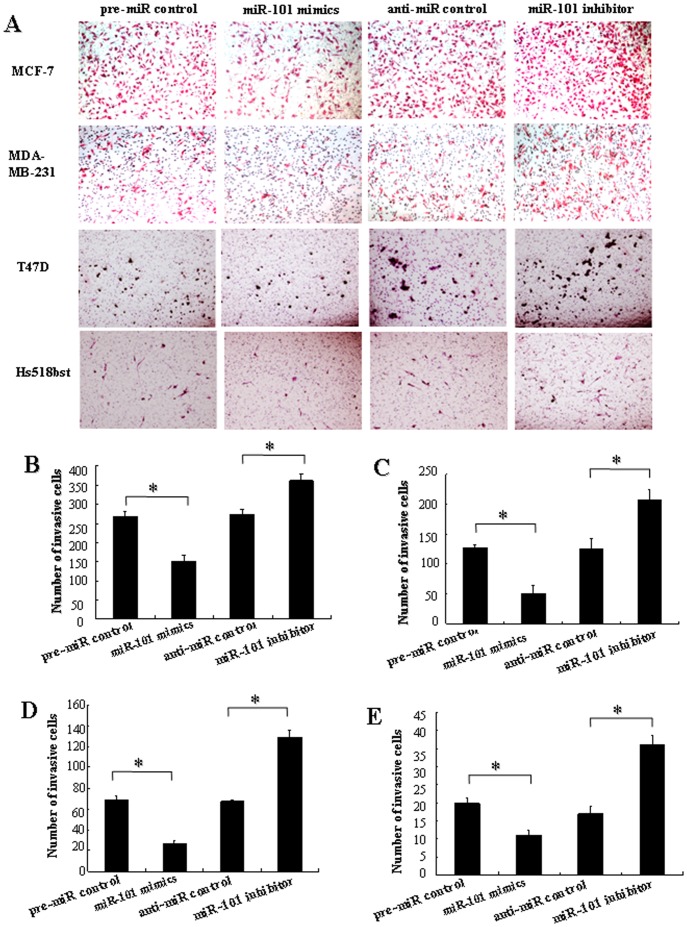
The effects of *miR-101* on cell invasion of breast cells. The cell invasion was quantified by counting the amount of cells passing through the membrane from five different fields per sample at 100× selected in a random manner after 24 h of incubation. A show the representative photomicrographs of cells, which were stained with haematoxylin and eosin, passing through the membrane at ×100 original magnification. Histogram B–E represents the number of invaded cells in MCF-7, MDA-MB-231, T47D and Hs518bst cells, respectively. Data are expressed as the mean numbers of independent triplicate experiments. *P<0.05.

In order to further research the role of *miR-101* in controlling the metastasis of breast cells, we analyzed the effects of *miR-101* on the migratory and invasive behavior of breast cancer cells and non-malignant mammary gland epithelial cell Hs518bst (Figure 6 and 7). The results showed that the migration capacity in MCF-7, MDA-MB-231, T47D and Hs518bst cells transfected with *miR-101* mimics was significantly lower than that transfected with pre-miR control (*P<*0.05). The migration capacity was significantly enhanced in MCF-7, MDA-MB-231, T47D and Hs518bst cells transfected with *miR-101* inhibitor compared with anti-miR control (*P<*0.05) (Figure 6). Additionally, the invasion assay indicated that the invasive capacity in MCF-7, MDA-MB-231, T47D and Hs518bst cells transfected with *miR-101* mimics was significantly inhibited compared with pre-miR control (*P<*0.05). While MCF-7, MDA-MB-231, T47D and Hs518bst cells were transfected with *miR-101* inhibitor, the invasive ability was obviously enhanced compared with anti-miR control (Figure 7). These fingdings suggest that the level of *miR-101* may be closely associated with the metastasis of breast cancer cells and non-malignant mammary gland epithelial cells.

### *Stmn1* is a Direct Target of *miR-101*

**Figure 8:**
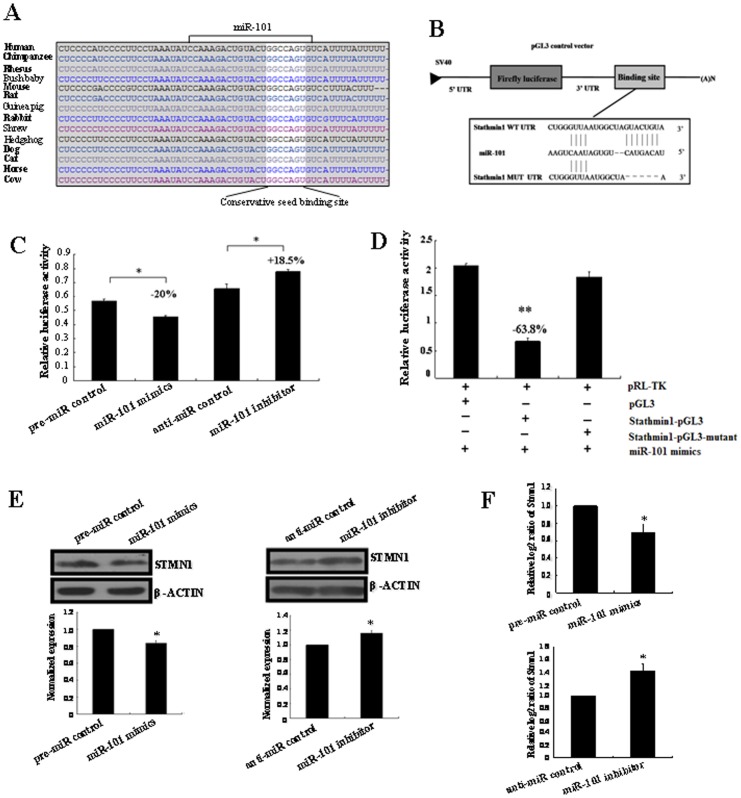
The prediction and confirmation of the *miR-101* target. (A) *MiR-101* binding sites in the 3′-UTR region of *Stmn1* in cross-species. (B) Schematic diagram for constructing the *miR-101* binding site into pGL3 control vector. (C) Confirmation of the target gene of *miR-101*. T47D cells were co-transfected with pre-miR control, *miR-101* mimics, anti-miR control or *miR-101* inhibitor and Stmn1-pGL3 for dual-luciferase assay. PRL-TK containing Renilla luciferase was co-transfected with 3′-UTR of *Stmn1* for data normalization. (D) Mutation analysis of the *miR-101* binding site. When the binding site of *miR-101* in the 3′-UTR of *Stmn1* was completely deleted (Stmn1-pGL3-Mutant), the luciferase activity was significantly decreased in T47D cells co-transfected with *miR-101* mimics and Stmn1-pGL3 compared with Stmn1-pGL3-Mutant or pGL3. (E) STMN1 protein level in *miR-101* mimics or inhibitor-treated T47D cells was detected by western blot. The bands were analyzed using the Quantity One analyzing system (Bio-Rad Laboratory inc.). The expression of β-ACTIN served as an internal control. The black histogram represents the optical densities of the signals quantified by densitometric analysis and represented as STMN1 intensity/β-ACTIN intensity to normalize for gel loading and transfer. (F) The expression of *Stmn1* mRNA in *miR-101* mimics and inhibitor-treated T47D cells were detected by qRT-PCR. β-actin serves as an internal reference among different samples and helps normalize for experimental error. The *y*-axis displays the relative log2 ratio of *Stmn1* normalized by β-actin. *P<0.05. **P<0.01.

**Figure 9:**
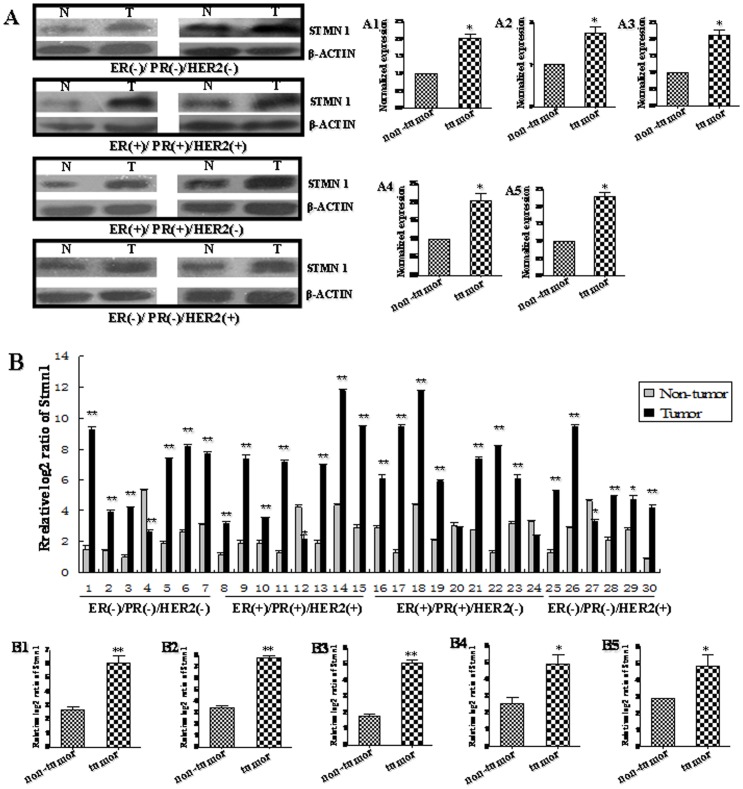
Up-regulation of *Stmn1* in human breast cancer tissues. We pooled two patients' cancer tissues as a sample. (A) The protein level of STMN1 in human breast cancer tissues was detected by western blot. The bands were analyzed using the Quantity One analyzing system (Bio-Rad Laboratory inc.). The expression of β-ACTIN served as an internal control. The black histograms represent the expression level of STMN1 in breast cancer tissues of all (A1), ER/PR−,HER2−(A2), ER/PR+, HER2+ (A3), ER/PR+, HER2− (A4) and ER/PR−,HER2+(A5) and expressed as STMN1 intensity/β-ACTIN intensity to normalize for gel loading and transfer. N represents non-tumor tissues, and T represents tumor tissues. (B) The mRNA level of *Stmn1* in human breast cancer tissues and adjacent normal breast tissues was detected by qRT-PCR. The expression level of *Stmn1* was obviously increased in 83.33% individual sample. Statistical analyses were performed to analyze the overall trend of *Stmn1* in human breast cancer tissues of all (B1), ER/PR−,HER2− (B2), ER/PR+, HER2+ (B3), ER/PR+, HER2− (B4) and ER/PR−, HER2+ (B5). β-actin serves as an internal reference. The *y*-axis displays the relative log2 ratio of *Stmn1* normalized to β-actin. *P<0.05, **P<0.01.

**Figure 10:**
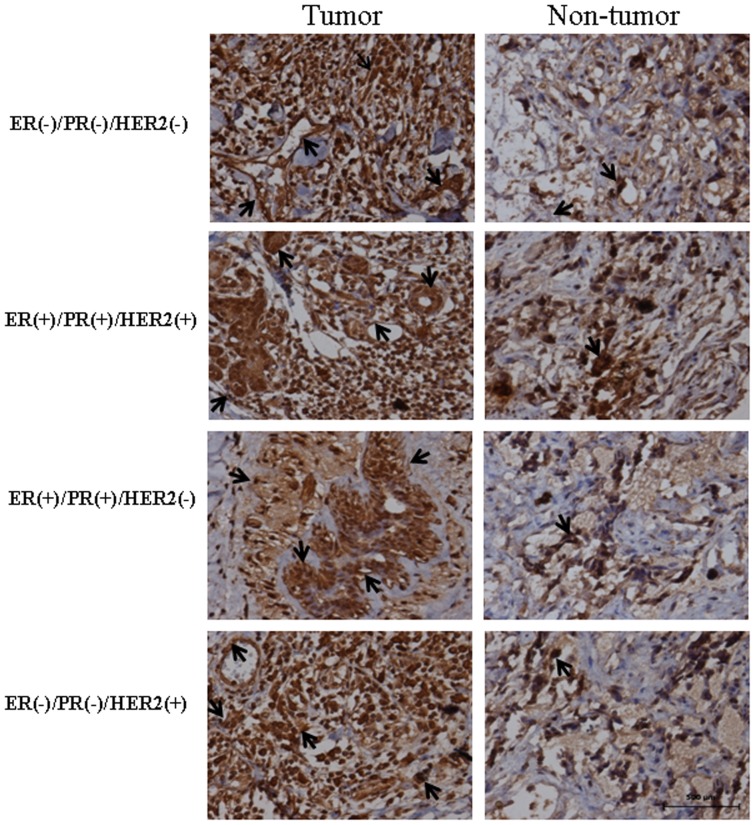
The distribution of STMN1 in breast tissues. The location of *miR-101* in breast cancer tissues and adjacent normal breast tissues was analyzed by immunohistochemistry using anti-STMN1 antibody. The sections were incubated with normal goat serum as negative control (Figure S1B). The stain was developed with DAB and the nuclei were stained with haematoxylin. Black arrows indicate positive immunoreaction. The scale bar indicated a distance of 500 µm.

To figure out the molecular mechanisms by which *miR-101* may perform in breast carcinogenesis, we looked for its target genes. An online search of *miR-101* targets by Targetscan, PicTar and miRanda provided a large number of putative miRNA targets. Among them, we focused on *Stmn1* for the following reasons: (*i*) Targetscan, PicTar and miRanda prediction showed that there was a *miR-101* responsive element in 3′-UTR of *Stmn1*, which is the highly conserved domain among different species (Figure 8A). (*ii*) It was reported that *Stmn1* plays an oncogenic role and was associated with polyploidy, tumor-cell invasion, early recurrence, and poor prognosis [19], [20]. Our previous study found that *Stmn1* was up-regulated in arsenic-induced angiogenesis by miRNA microarray analysis [21]. More importantly, *Stmn1* was observed to overexpress in human breast cancer tissues, which was not restricted to a specific sub-group of breast carcinoma [22]–[24]. Our results showed that the decrease of *miR-101* was also not restricted to a specific subtype of breast carcer. (*iii*) In this study, we found that the level of *Stmn1* expression was up-regulated (Figure 9 and 10), consistent with previous reports [22]–[24], and *miR-101* was down-regulated in human breast cancer tissues (Figure 1). Thus, an evident inverse relationship was showed between the expression of *miR-101* and *Stmn1* in human breast cancer.

To validate whether *Stmn1* is indeed the target gene of *miR-101*, a human *Stmn1* 3′-UTR fragment containing wild-type was cloned into the downstream of the firefly luciferase reporter gene in the pGL3 control vector (designated as Stmn1-pGL3) for the dual-luciferase assay (Figure 8B). T47D cells were co-transfected with Stmn1-pGL3 and *miR-101* mimics or inhibitor (Figure 8C). Compared with the pre-miR control, the luciferase activity was significantly suppressed by the *miR-101* mimics, about 20.00% (*P*<0.05). Furthermore, the luciferase activity was significantly up-regulated by the *miR-101* inhibitor compared with the control, about 18.50% (*P*<0.05). These results indicate that *miR-101* affects the binding of *miR-101* and 3′-UTR of *Stmn1*, leading to the change of *miR-101* translation.

Base mutation of seed sequence was also conducted to further confirm the binding site for *miR-101* (Figure 8D). Deleting putative *miR-101* binding region in the 3′-UTR of *Stmn1* (designated as Stmn1-pGL3-Mutant) and pGL3 empty vector were used as control, respectively. The histogram in Figure 8D showed that the enzyme activity was reduced about 63.80% in cells co-transfected with *miR-101* mimics and Stmn1-pGL3 compared with *S*tmn1-pGL3-Mutant or pGL3 (*P*<0.01). These data indicate that *miR-101* may suppress gene expression through binding to seed sequence at the 3′-UTR of *Stmn1*, and *Stmn1* may be a direct target of *miR-101*.

### *Mir-101* Regulates Endogenous *Stmn1* Expression in Breast Cancer Cells *in vitro*

Although *Stmn1* was identified as a target gene for *miR-101*, it was unknown whether *miR-101* could regulate endogenous *Stmn1* expression. T47D cells were transfected with *miR-101* mimics or inhibitor to see whether the dysregulation of *miR-101* expression affected endogenous *Stmn1* expression. Compared with corresponding control, the level of STMN1 protein was significantly down-regulated by *miR-101* mimics (*P*<0.05) and up-regulated by *miR-101* inhibitor (*P*<0.05) (Figure 8E). Additionally, the level of *Stmn1* mRNA detected by qRT-PCR was significantly decreased by *miR-101* mimics (*P*<0.05) and increased by *miR-101* inhibitor (*P*<0.05) compared with corresponding control (Figure 8F). These results showed that the expression of endogenous *Stmn1* mRNA and protein were regulated by *miR-101*.

### *Stmn1* Was Involved in the Effect of *mir-101* on Cell Growth and Metastasis

**Figure 11:**
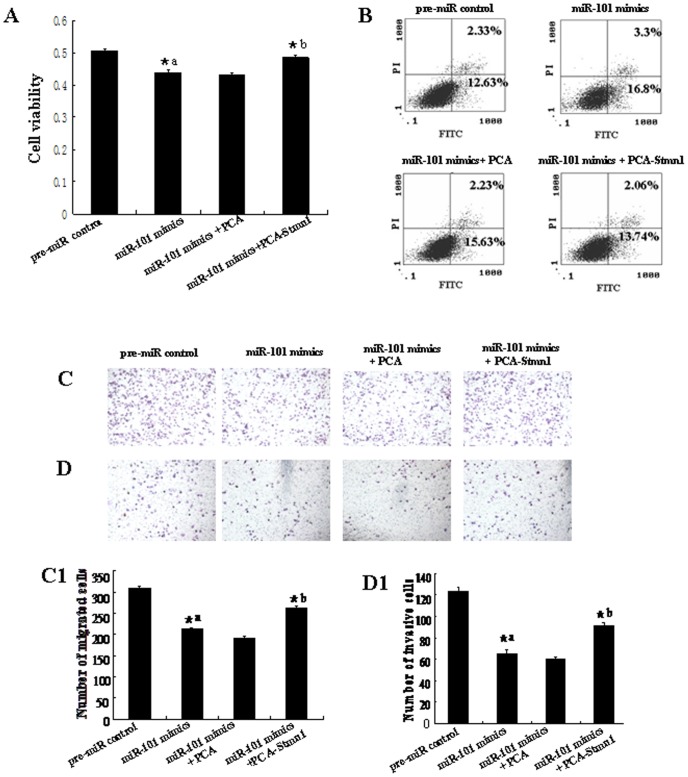
Overexpresson of *Stmn1* partially restored the inhibition of cell growth and metastasis induced by up-regulation of *miR-101*. PCA-Stmn1 or empty vector control PCA were transfected into MDA-MB-231 with the lipofectamine 2000. Cell proliferation was determined by MTT assay (A). All experiments were performed at least three times and calculated cell proliferation by OD value at A570. Cell apoptosis were detected by flow cytometry (B). Cells (0.5×10^6^ cells/ml) were seeded Transwells for cell migration assay (C) or matrigel-coated Transwells for cell invasion assay (D). The detailed explanation for apoptosis, cell migration and invasion assay was referred to the legends for figure 5–7. *^a^: pre-miR control vs *miR-101* mimics, *P*<0.05; *^b^: *miR-101* mimics vs *miR-101* mimics+PCA or *miR-101* mimics+PCA-Stmn1, *P*<0.05.

**Figure 12:**
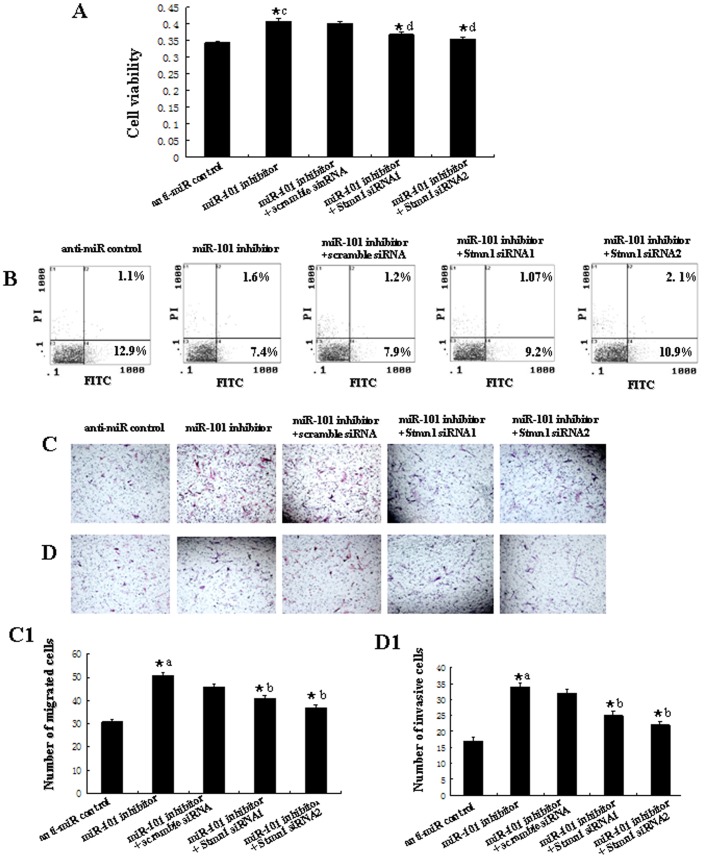
Knockdown of *Stmn1* attenuates the down-regulation of *miR-101-*mediated enhancement of cell growth and metastasis. *Stmn1* siRNA1, siRNA2 or scramble siRNA control were transfected into Hs518bst with the lipofectamine 2000. Cell proliferation was determined by MTT assay (A). Cell apoptosis were detected by flow cytometry (B). Cells (0.5×10^6^ cells/ml) were seeded Transwells for cell migration assay (C) or matrigel-coated Transwells for cell invasion assay (D). The detailed explanation for cell proliferation, apoptosis, cell migration and invasion assay was referred to the legends for figure 11. *^a^: anti-miR control vs *miR-101* inhibitor, *P*<0.05; *^b^: *miR-101* inhibitor vs *miR-101* inhibitor+Scramble siRNA, *miR-101* inhibitor+*Stmn1* siRNA1 or *miR-101* inhibitor+*Stmn1* siRNA2, *P*<0.05.

To further test whether *miR-101* may execute tumor-suppressive functions by targeting *stmn1*, we investigated the effect of *Stmn1* on *miR-101*-mediated cell behavior. The effect of *Stmn1* constructs on the expression level of *Stmn1* was detected by western blot and qRT-PCR. *Stmn1* expression level was markedly enhanced by PCA-Stmn1 (*P<*0.01), and remarkably decreased by *Stmn1* siRNA (*P<*0.01; Figure S4). MDA-MB-231 cells that expressed low level of *miR-101* were co-transfected with PCA-Stmn1 and *miR-101* mimics, which showed higher capacity of proliferation, migration and invasion and lower level of apoptosis than that transfected with *miR-101* mimics (Figure 11), implying that *miR-101* overexpression-mediated the suppression of cell growth and metastasis was partially rehabilitated by up-regulation of Stmn1. Additionally, *Stmn1* siRNA and *miR-101* inhibitor and were co-transfected into Hs518bst that expressed high level of *miR-101*. We found that cell proliferation, migration and invasion capacity was lower and apoptosis level was higher in cells co-transfected by *Stmn1* siRNA and *miR-101* inhibitor than that transfected by *miR-101* inhibitor (Figure 12), displaying that *miR-101* low expression-mediated the promotion of cell growth and metastasis was partially attenuated by knockdown of *Stmn1*. Taken together, these results indicate that *miR-101* executes functions in breast cancer cells partially by targeting *Stmn1*.

### Up-Regulation of *Stmn1* Expression in Human Breast Cancer Tissues

To further explore the expression profiles of *miR-101* target gene, *Stmn1*, *in vivo*, the protein and mRNA level of *Stmn1* in breast cancer tissues were detected by western blot and qRT-PCR (Figure 9). In this study, we pooled two patients' cancer tissues as a sample. The results of western blot showed that the protein level of *Stmn1* was significantly increased in all breast cancer tissues and four subtypes, respectively, compared with adjacent normal breast tissues (*P*<0.05) (Figure 9A). Additionally, the mRNA level of *Stmn1* was remarkably increased in 83.33% (25/30 samples) of breast cancer tissues (Figure 9B). In general, the expression of *Stmn1* was significantly higher in all human breast cancer tissues than that in adjacent normal breast tissues (*P<*0.01; Figure 9B1). The results from the analysis of the trend of *Stmn1* expression in four different subtypes of breast cancer tissues were consistent with that in all breast cancer tissues. *Stmn1* was overexpressed in breast cancer samples of 85.71% (6/7 samples) of triple negative, 87.50% (7/8 samples) of ER/PR+, HER2+, 77.78% (7/9 samples) ER/PR+, HER2− and 83.33% (5/6 samples) of ER/PR−, HER2+, when compared with that in adjacent normal breast tissues, respectively (Figure 9B). The overall expression of *Stmn1* was significantly enhanced in human breast cancer tissues of triple negative (*P<*0.01), ER/PR+, HER2+ (*P<*0.01), ER/PR+, HER2− (*P<*0.05) and ER/PR−, HER2+ (*P<*0.05) (Figure 9B2–5). The expression pattern of *Stmn1* was opposite with *miR-101* in breast cancer tissues, indicating that *miR-101* may be involved in regulating breast carcinogenesis by interacting with *Stmn1 in vivo*.

The distribution of STMN1 in breast cancer tissues and adjacent normal breast tissues was determined by immunohistochemistry (Figure 10). STMN1 was mildly detected in normal breast tissues. Strong signals of STMN1 were found in four subtypes of breast tissues. STMN1 was mainly localized in breast epithelial, stromal, vascular endothelial and cancer cells. There was no immunoreaction in the sections of breast cancer tissues incubated with normal goat serum as negative control (Figure S1B).

## Discussion

To our knowledge, our study provides the first piece of evidence that *miR-101* is involved in breast carcinogenesis, although many studies have showed that down-regulation of *miR-101* is associated with in various cancers originated from lung, gastric, ovary, colon cancer, prostate and gliolastoma [11], [13], [14], [25]–[28].

In this study, we found that *miR-101* expression was significantly down-regulated in ER/PR−, HER2−,ER/PR+, HER2+,ER/PR+, HER2− and ER/PR−, HER2+ breast cancer tissues compared with that in adjacent normal breast tissues. It seems that the down regulation of *miR-101* is sensitive to different subtypes of breast cancer, suggesting that down-regulation of *miR-101* is not restricted to a specific subtype of breast carcinoma and *miR-101* may be involved in the occurrence of breast cancer.

Therefore, here, we further explored the biological functions of *miR-101* in breast cancer. We found that re-expression of *miR-101* in breast cancer cell lines and non-malignant mammary gland epithelial cells inhibited cells proliferation, migration and invasion, but promoted apoptosis, and vice versa. Zhang et al. reported that the down-regulation of *miR-101* in non-small cell lung cancer promoted cell proliferation and invasion and inhibited paclitaxel-induced apoptosis [14]. Wang et al. found that ectopic expression of *miR-101* could inhibit proliferation, migration and invasion of gastric cancer cells *in vitro* [15]. The role of *miR-101* in breast cancer cells was coincident with that in other cancers. All these results further confirm that *miR-101* functions as a tumor suppressor.

It is generally accepted that miRNAs exert their function through regulating the expression of their downstream target genes. In recent years, many studies have focused on the role of miRNAs in cellular growth, differentiation and apoptosis of cancer cells through interactions between miRNAs and their target genes. *Stmn1*, also known as p17, p18, p19, 19K, metablastin, oncoprotein 18, LAP18 and Op18, is a 19kDa cytosolic protein, composed of 149 amino acids organized into four domains (I–IV). It is the first discovered member of a family of phylogenetically related microtubule-destabilizing phosphoproteins, which plays important roles in construction and function of the mitotic spindle [29], [30]. As a cytosolic phosphoprotein, *Stmn1* is proposed to act as a relay integrating diverse cell signaling pathways, especially during the control of cell growth and differentiation [22]. It has been reported that *Stmn1* is overexpressed in many human malignancies, such as leukemia, lymphoma, neuroblastoma, ovarian, prostatic, breast and lung cancers [31]. The expression of STMN1 in cancer cells has been associated with their proliferation and metastasis [32], [33]. Oishi et al. reported that STMN1 protein levels were significantly higher in breast cancer patients with poor prognosis than that with good prognosis, indicating that *Stmn1* immnunohistostaining is a potential method for predicting the outcome of breast cancer patients with supraclavicular lymph node metastasis [34]. These studies suggest that *Stmn1* may play critical roles in the progress of breast cancer, although the specific molecular mechanism is still unclear.

In our study, we identified *Stmn1* as a target of *miR-101* and three lines of evidence supported this finding. First, there is a *miR-101* binding site in the 3′-UTR of *Stmn1* predicted by Targetscan, PicTar and miRanda. Second, overexpression of *miR-101* reduced the protein and mRNA level of *Stmn1* and knockdown of *miR-101* promoted the expression of *Stmn1 in vitro*. A reverse correlation was also found between the level of *miR-101* and *Stmn1* in human breast cancer tissues. Third, *Stmn1* 3′-UTR-mediated luciferase activity is specifically responsive to transfection of *miR-101* mimics and inhibitor. Moreover, base mutation of seed sequence in the 3′-UTR of *Stmn1* significantly reduced the binding capacity of *miR-101* and 3′-UTR of *Stmn1*. The overexpression of *Stmn1* in human breast cancer tissues correlated with general predictive factors and not restricted to a specific sub-group of breast carcinoma [22]–[24]. However, we found that the expression of *miR-101* was obviously decreased in different subtypes of human breast cancer tissues and also not restricted to a specific subtype of breast carcinoma. Additionally, we found that the up-regulation of *miR-101*-mediated the inhibition of cell growth and metastasis was partially recovered by overexpresson of *Stmn1*. Knockdown of *Stmn1* weakened the effect of down-regulation of *miR-101* on cell growth and metastasis. Thus, we speculated that *miR-101* may exert its tumor suppressor function at least in part by regulating the expression of *Stmn1* during the occurrence of breast cancer.

Interestingly, our results showed that the expression of *miR-101* was also inversely correlated with the *Stmn1* mRNA level not only in breast cancer cells, but also in breast cancer tissues. Initially, miRNAs were thought to function mainly by suppressing mRNA translation [35]. However, two recent studies combined proteomics and microarrays to reveal that the changes in protein expression mediated by a miRNA were usually associated with the alteration of mRNA level, suggesting that mRNA degradation may be the major component of mammalian miRNA repression [36], [37]. Our results were also consistent with this point of view. In fact, a specific miRNA has the potential to target multiple genes, *miR-101* perform its function may through cooperative regulation of its other target genes. In the future, other targets of *miR-101* besides *Stmn1* still need to be identified in breast cancer.

In conclusion, *miR-101* is significantly down-regulated in different subtypes of human breast cancer tissues, which promotes cellular proliferation, migration and invasion, but inhibits apoptosis in breast cancer cells. These effects may at least partially due to direct repression of *Stmn1* by *miR-101*. Our data not only suggest an important role of *miR-101* in breast carcinogenesis, but also imply that therapeutic strategies aimed at restoration of *miR-101* expression may be beneficial to patients with breast cancer.

## Materials and Methods

### Tissue Samples and Breast Cell Lines

Human breast cancer tumors and adjacent non-tumor tissues were obtained from patients at the Third Affiliated Hospital of Harbin Medical University, Harbin, China and Tangshan tumor Hospital, China. The clinicopathologic characteristics of patients and breast tumors are summarized in Table 1. All patients provided informed consent for the use of their tissues before surgery. After surgical removal, the tissues were frozen immediately in liquid nitrogen and stored at −80°C until used. Sixty pairs of human breast cancer tissues and adjacent normal breast tissues were collected. We pooled two patients' cancer tissues as a sample (30 samples for 60 pairs) for the analysis of qRT-PCR and western blot. This study was approved by the Ethics Committee of National Research Institute for Family Planning (No.2011-12). The ethics committee specifically approved that procedure and what measures were taken to document the process. The collection of tissues followed the procedures that are in accordance with the ethical standards as formulated in the Helsinki Declaration.

Human breast carcinoma cell lines T47D (ER alpha-positive), MCF-7 (ER alpha -positive) MDA-MB-231 (ER alpha-negative) and Hs518bst (non-malignant mammary gland epithelial cell) were obtained from shanghai institute of biochemistry and cell biology (Shanghai, China), and cultured in Dulbecco's Modified Eagle Medium (DMEM) containing 10% fetal bovine serum (FBS), 100 IU/ml penicillin and 10 mg/mL streptomycin. All cells were maintained at 37°C under an atmosphere of 5% CO_2_.

### Plasmid Construction and Transfection

The *Stmn1* 3′-UTR and *Stmn1* 3′-UTR-mutant sequences were amplified by PCR from human genomic DNA using the primers in Table 2. After being double digested with *Spe* I and *Pst* I, the PCR product was cloned into pGL3 control vector (Invitrogen, Carlsbad, CA, USA). The coding region of *STMN1* sequence was amplified by RT-PCR from total mRNA of human T47D cells using the primers in Table 2. After being double digested with *Xba* I and *Sac* I, the PCR product was cloned into PCAGGS-IRES-EGFP (PCA) control vector, designated PCA-Stmn1. All the constructs were verified by DNA sequencing.

**Table 2:** Primer sequences.

Gene name	Primer sequence	Accession number	Size and location	Application
*Stathmin1* 3′- UTR (sense)	Forward/*Spe I*: 5′-GGACTAGTGAACTGACTTTCTCCCCATCCC-3′	NM-005563	444 bp (636–1079)	PCR
	Reverse/*Pst I*: 5′-AACTGCAGGCCACCAACAGCACTGTG-3′			
*Stathmin1* 3′-UTR (deleting binding region)	The first part of sequence	NM-005563	276 bp (636–911)	PCR
	Forward/*Spe I*: 5′-GGACTAGTGAACTGACTTTCTCCCCATCCC-3′			
	Reverse::5′-TAGCCATTAACCCAGTACACCAAG-3′			
	The second part of sequence	NM-005563	161 bp (919–1079)	PCR
	Forward: 5′-CTTGGTGTACTGGGTTAATGGCTAATTGGCTCTGTGAAAACAT-3′			
	Reverse/*Pst I*: 5′-AACTGCAGGCCACCAACAGCACTGTG-3′			
*STMN1* (coding region)	Forward: 5′-GCTCTAGAGCATGGCTTCTTCTGATATCCAGGTG-3′	NM-005563.3	450 bp (177–626)	RT-PCR
	Reverse: 5′-CGAGCTCGCTAGTGATGGTGAGAGAGATCAGAC-3′			
*Stathmin1*	Forward: 5′-TGGCAGAAGAGAAACTGACCCACA-3′	NM-005563	151 bp (461–611)	qRT-PCR
	Reverse:: 5′-TCTCGTCAGCAGGGTCTTTGGATT-3′			
*β*-*actin*	Forward: 5′-AGGACGACGAATCTTCTCAATGGG-3′	NM-001100	91 bp (1270–1386)	qRT-PCR
	Reverse::5′-GTTTACGATGGCAGCAACGGAAGT-3′			

The *miR-101* mimics, pre-miR control, *miR-101* inhibitor, anti-miR control, scramble siRNA control and *Stmn1* siRNA were synthesized by GenePharma (GenePharma Co.,Ltd, Shanghai, China), was transfected into cells by the lipofectamine 2000 (Invitrogen, Carlsbad, CA, USA) essentially as described as the manufacture's instruction. The final concentration is 50 µM. Specific siRNAs for scramble and Stmn1 were synthesized as a duplex with the following sequence: scramble siRNA, UUCUCCGAACGUGUCACGU-dTdT, *Stmn1* siRNA1, AAGAGA AACUGACCCACAA-dTdT, *Stmn1* siRNA2, UAAAGAGAACCGAGAGGCA -Dtdt.

### Quantitative Reverse-Transcriptase Polymerase Chain Reaction (qRT-PCR) Analysis of miRNA and mRNA

Quantitive RT-PCR analysis was used to determine the relative expression level of *miR-101* and *Stmn1*. Total RNA was extracted from tissues and cells, using Trizol (Invitrogen, Carlsbad, CA, USA) according to the manufacture's instructions. The expression level of *miR-101* was detected by TaqMan miRNA RT-Real Time PCR. Single-stranded cDNA was synthesized by using TaqMan MicroRNA Reverse Transcription Kit (Applied Biosystems, Foster City, CA, USA) and then amplified by using TaqMan Universal PCR Master Mix (Applied Biosystems, Foster City, CA, USA) together with miRNA-specific TaqMan MGB probes: *miR-101* and *U6* (Applied Biosystems, Foster City, CA, USA). The U6 snoRNA was used for normalization. The expression level of *Stmn1* was detected by RT-Real-time PCR. Total RNA (1 µg) was used as a template for reverse transcription using the iScript™ cDNA Synthesis Kit (Bio-Rad, Hercules, CA, USA). The mRNA level of *stmn1* and glyceraldehyde-3-phosphate dehydrogenase (*Gapdh*) was amplified by a FastStart Universal SYBR Green Master (Roche, Mannheim, Germany) and StepOne™ Real-Time PCR System (Applied Biosystems, Foster City, CA, USA) using the primers in Table 2. The quantification was normalized to an endogenous control *Gapdh.* Each sample in each group was measured in triplicate and the experiment was repeated at least three times for the detection of *miR-101* and *Stmn1*.

### *In situ* Hybridization of miR-101 with DIG-labeled LNA Probe

The sections (4 µm) of the breast cancer tissues and adjacent normal breast tissues were treated with proteinase K (20 µg/ml) for 15 min and refixed in 4% PFA for 15 min. After acetylation with 0.25% acetic anhydride in 0.1 M triethanolamine, pH 8.0 for 10 min, sections were prehybridized with hybridization buffer (Roche, Mannheim, Germany) at 40°C for 2 h and then hybridized with digoxigenin (DIG)-labeled LNA-miR-101 probe (LNA-miR-101 sequences: 5′-ttCagTtatCacaGtaCtgTa-3′) at 40°C overnight. The sections were then incubated in buffer containing anti-DIG-antibody (Roche, Mannheim, Germany) 2 h at 37°C, followed by staining with NBT and BCIP (Promega, Madison, WI, USA). The sections of normal breast tissues were hybridized with DIG-labeled LNA-scrambled probe (LNA-scrambled sequences: 5′-caTtaAtgTcGgaCaaCtcAat-3′) as negative control [22]. Samples were viewed under Nikon TE 2000-U microscope (NIKON, Tokyo, Japan).

### Cell Proliferation Assay

Cell proliferation was estimated with an MTS assay. T47D, MCF-7, MDA-MB-231 and Hs518bst cells were seeded in 96-well plates at low density (5000 cells per well) in DMEM culture, and allowed to attach overnight. The cells were then transfected with *miR-101* mimcs, pre-miR control, *miR-101* inhibitor and anti-miR control, respectively. Each treatment group was repeated 3 wells. Twenty microlitres MTT (5 mg/ml; Sigma-Aldrich, St. Louis, MO, USA) were added to each well 48 h after transfection, and the cells were incubated for further 4 h. Media was then removed and the 150 µl dimethyl sulfoxide (Sigma-Aldrich, St. Louis, MO, USA) was added in each well. Absorbance was recorded at A570 nm with a 96-well plate reader (Bio-Rad 3550). The experiment has been repeated for three times and the results were described as a ratio of A570 nm with *miR-101* mimcs or *miR-101* inhibitor vs its corresponding control.

### Flow Cytometry Analysis

Cells apoptosis was performed with flow cytometry analysis by Annexin V-FLUOS staining Kit (Roche, Mannheim, Germany), containing 2.5 µl annexin V-fluorescein isothiocyanate (FITC) stock and 5 µl 20 µg/ml phosphatidylinositol (PI) to determine the phosphatidylserine exposure on the outer plasma membrane. After incubation for 15 min at room temperature in a light-protected area, the specimens were quantified by flow cytometry (BD Biosciences, San Jose CA, USA), acquiring 8,000 events. Each treatment was repeated twice within an experiment. The experiment has been repeated for three times. In order to adjust fluorescence compensation to remove the overlap spectral overlap and set gating, unstained cells, cells stained with PI alone or annexin V/FITC alone were detected by flow cytometry. The total live cell population was gated by detecting unstained cells. The apoptosis cell population was gated by detecting cells stained with annexin V/FITC alone and the dead cell population by detecting cells stained with PI alone. The analysis results of these cells in flow cytometry were used to set the location of the Cross Gate. This detection was performed in every cell line.

### *In vitro* Migration and Invasion Assays

MCF-7, MDA-MB-231, T47D and Hs518bst were infected with the *miR-101* mimics, pre-miR control, *miR-101* inhibitor or anti-miR control, respectively. The infected cells were harvested and subjected to the following assays, 48 h after transfection. For migration assays, the infected cells (0.5×10^6^ cells/ml) were seeded in the top of an 8.0-µm-pore membrane chamber (Corning Costar Corp., Cambridge, MA, USA). Following a 17 h incubation period, cells that passed through the membrane to attach to the bottom of membrane were fixed and stained with hematoxylin and eosin (Sigma-Aldrich, St. Louis, MO, USA). Cells were scraped and removed from the top of chamber. Membranes were mounted on cover slides, and cells were counted. The cell migration was quantified by counting the amount of cells passing through the pores from five different fields per sample at 100× selected in a random manner. For invasion assays, matrigel (BD Biosciences, San Jose CA, USA) diluted to 1 mg/ml in serum free-cold cell culture media was added in the top of an 8.0-µm-pore membrane chamber (Corning Costar Corp., Cambridge, MA, USA) and incubated at 37°C for 4 h until the matrigel solidified. Cells (0.5×10^6^ cells/ml) were seeded on the top chamber with matrigel-coated membrane. After 24 h of incubation, cells that had invaded to the lower chamber were fixed and stained with hematoxylin and eosin (Sigma-Aldrich, St. Louis, MO, USA). Cells were scraped and removed from the top of chamber. Membranes were mounted on cover slides, and cells were counted. The cell invasion was quantified by counting the amount of cells passing through the membrane from five different fields per sample at 100× selected in a random manner.

### Dual-luciferase Activity Assay

To generate 3′-UTR luciferase reporter, partial sequence of the 3′-UTR from *Stmn1* were cloned into the downstream of the firefly luciferase gene in pGL3-Control Vector (Promega, Madison, WI, USA). Deleting *miR-101* target sites in the 3′-UTR of *Stmn1* was used as control. pRL-TK containing Renilla luciferase was co-transfected for data normalization. For luciferase reporter assays, T47D cells were seeded in 48-well plates and allowed to attach overnight, then transfected by using lipofectamine 2000 (Invitrogen, Carlsbad, CA, USA). Two days later, cells were harvested and assayed with the dual-luciferase assay (Promega, Madison, WI, USA). Each treatment was performed in triplicate in three independent experiments. The results were expressed as relative luciferase activity (Firefly LUC/Renilla LUC).

### Western Blot

Cell protein lysates were boiled in SDS/β-mercaptoethanol sample buffer, and 40 µg samples were loaded into each lane of 12% polyacrylamide gels. The proteins were separated by electrophoresis, and transferred to polyvinylidene fluoride membrane (PVDF) (Amersham Pharmacia Biotech, St Albans, Herts, UK) by electrophoretic transfer. The membrane was incubated with rabbit anti-STMN1 polyclonal antibody (Cell Signaling Technology, Inc., Danvers, MA, USA), anti-β-actin polyclonal antibody (Santa Cruz Biotechnology Inc., Santa Cruz, CA, USA) overnight at 4°C. The specific protein-antibody complex was detected by using horseradish peroxidase (HRP) conjugated goat anti-rabbit IgG (Jackson Immunoresearch Laboratories, Inc., West Grove, PA, USA). Detection by the chemiluminescence reaction was carried using the ECL kit (Millipore, Billerica, MA, USA). The β-actin signal was used as a loading control. The experiment has been repeated for at least three times. The bands were analyzed using Quantity One analyzing system (Bio-Rad, Hercules, CA, USA).

### Immunohistochemistry

Sections (4 µm) of the breast cancer tissues and adjacent normal breast tissues were deparaffinized in xylene and rehydrated in descending ethanol series. Antigen retrieval was accomplished through microwave irradiation of the sections in 10 mM sodium citrate buffer. Slides were incubated respectively with rabbit anti-STMN1 polyclonal antibody (Cell Signaling Technology, Inc., Danvers, MA, USA), then incubated with HRP-conjugated goat anti-rabbit IgG (Jackson Immunoresearch Laboratories, West Grove, PA, USA). The antibody stains were developed by addition of diaminobenzidine (DAB; Sigma-Aldrich, St. Louis, MO, USA) and the nuclei were stained with haematoxylin (Sigma-Aldrich, St. Louis, MO, USA). The sections were incubated with normal goat serum as negative control. Samples were viewed using under Nikon TE 2000-U microscope (NIKON, Tokyo, Japan).

### Statistical Analysis

All statistical analyses were performed using the SPSS 16.0 statistical software package. Data are presented as mean±SEM from at least three independent experiments. Multiple group comparisons were performed using one-way analysis of variance (ANOVA). Differences were considered statistically significant at *p*<0.05.

## Supporting Information

Figure S1 **The negative control for** ***in situ*** **hybridization and immunohistochemistry.** The sections of normal breast tissues were hybridized with DIG-labeled LNA scrambled miRNA probe as a negative control for *in situ* hybridization (A). The sections of breast cancer tissues were incubated with normal goat serum as negative control for immunohistochemistry (B).
(TIF)

Figure S2 **The endogenous** ***miR-101*** **level in breast cells.** RNA extracted from normal MCF-7, T47D, MDA-MB-231 and Hs518bst cells. The expression level of *miR-101* in these cells was detected by TaqMan miRNA RT-Real Time PCR. U6 serves as an internal reference among different samples and helps normalize for experimental error. The *y*-axis displays the relative log2 ratio of *miR-101* normalized by *U6*. **P<0.01.
(TIF)

Figure S3 **The analysis of growth curve in breast cell lines.** MCF-7, T47D, MDA-MB-231 and Hs518bst cells were respectively transfected by *miR-101* mimics or inhibitor and cell numbers at 12 h, 24 h, 36 h, 48 h, 60 h and 72 h was counted, respectively, in MCF-7 (A), T47D (B), MDA-MB-231 (C) and Hs518bst (D).
(TIF)

Figure S4 **The effect of** ***Stmn1*** **constructs on the expression level of** ***Stmn1*** **.** PCA or PCA-Stmn1 was transfected into MDA-MB-231 and scramble siRNA, Stmn1 siRNA1 or siRNA2 was transfected into Hs518bst. The STMN1 protein level was detected by western blot (A). The expression of β-ACTIN served as an internal control. The Stmn1 mRNA level was detected by RT-Real Time PCR (B). β-actin serves as an internal reference. **P<0.01.
(TIF)
