# Supplementary figures and images for: MiR-101 Is Involved in Human Breast Carcinogenesis by Targeting Stathmin1
Source: PLoS One. 2012 Oct 11;7(10):e46173. doi: 10.1371/journal.pone.0046173 (PMC3469601; doi:10.1371/journal.pone.0046173)

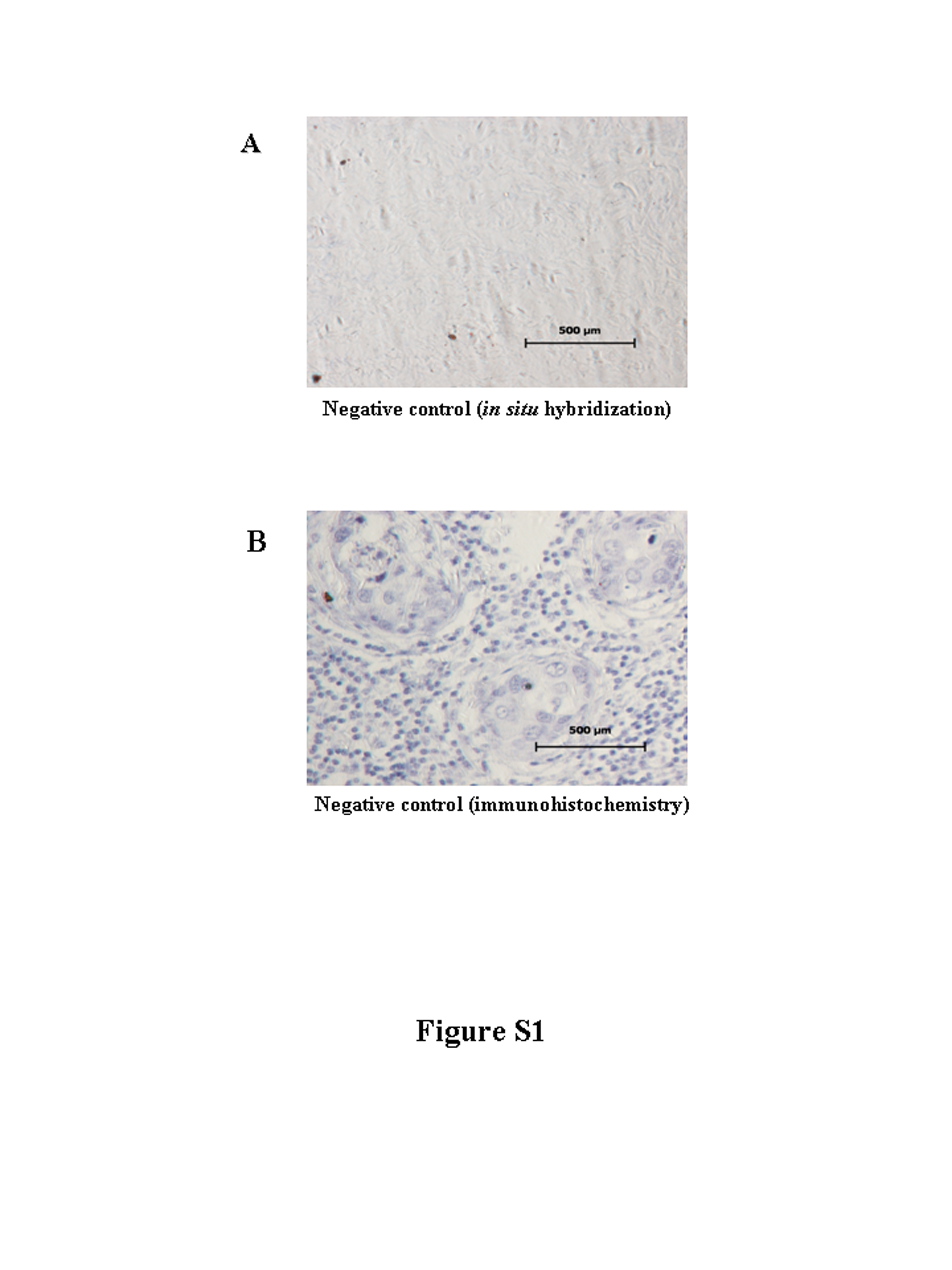

Supplement: Figure S1 — The negative control for in situ hybridization and immunohistochemistry. The sections of normal breast tissues were hybridized with DIG-labeled LNA scrambled miRNA probe as a negative control for in situ hybridization (A). The sections of breast cancer tissues were incubated with normal goat serum as negative control for immunohistochemistry (B). (TIF) [file pone.0046173.s001.tif]

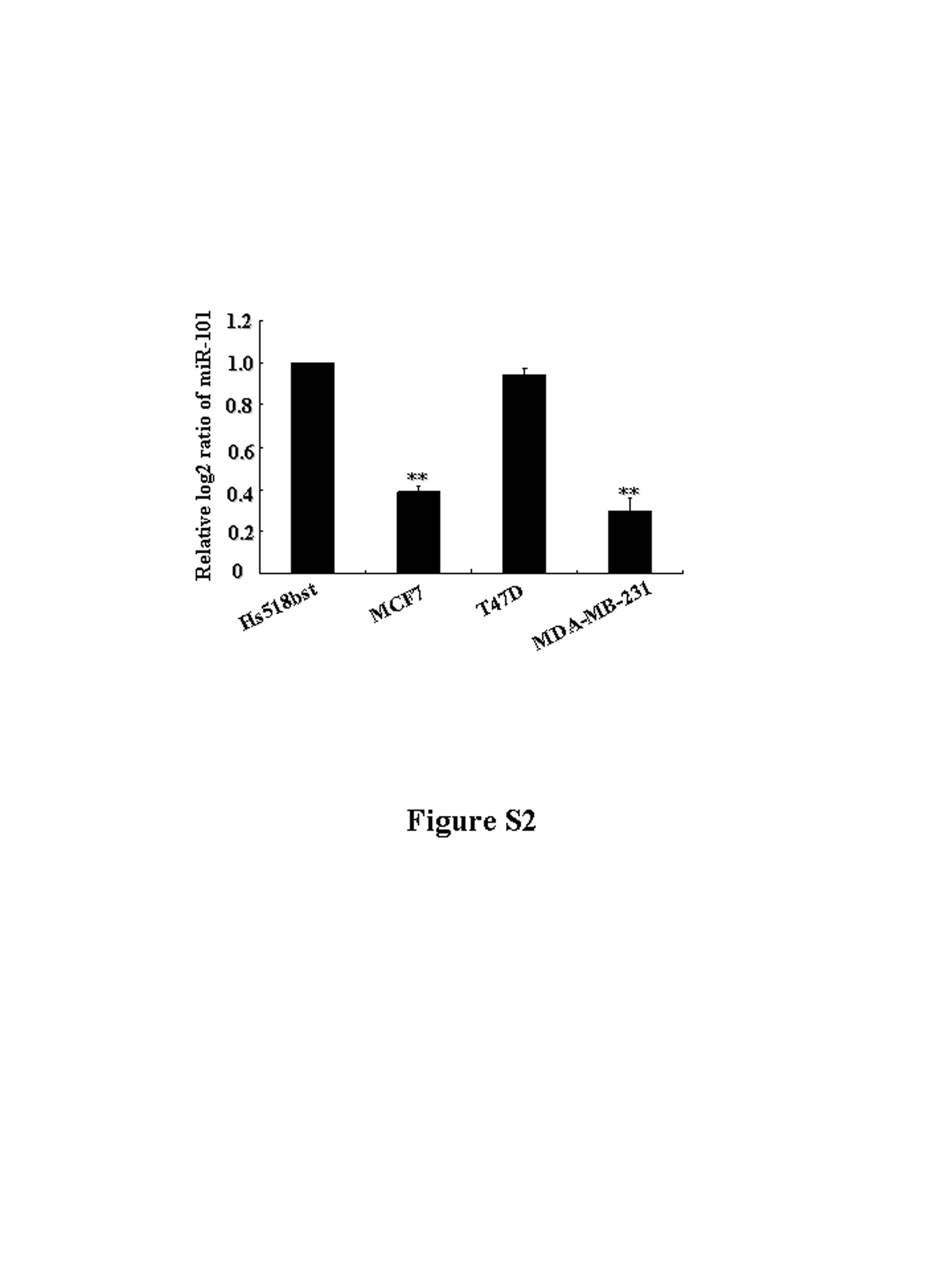

Supplement: Figure S2 — The endogenous miR-101 level in breast cells. RNA extracted from normal MCF-7, T47D, MDA-MB-231 and Hs518bst cells. The expression level of miR-101 in these cells was detected by TaqMan miRNA RT-Real Time PCR. U6 serves as an internal reference among different samples and helps normalize for experimental error. The y-axis displays the relative log2 ratio of miR-101 normalized by U6. **P<0.01. (TIF) [file pone.0046173.s002.tif]

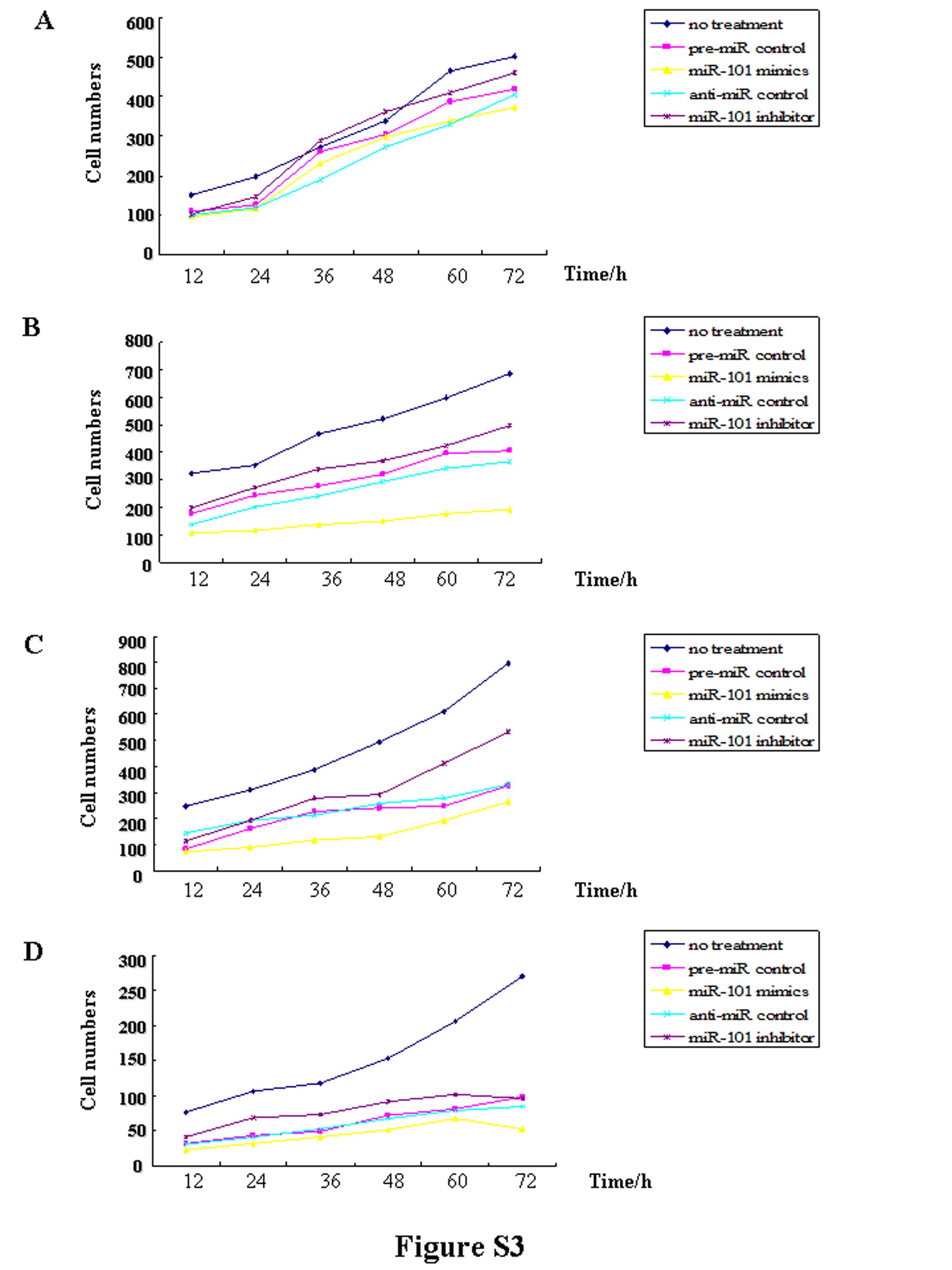

Supplement: Figure S3 — The analysis of growth curve in breast cell lines. MCF-7, T47D, MDA-MB-231 and Hs518bst cells were respectively transfected by miR-101 mimics or inhibitor and cell numbers at 12 h, 24 h, 36 h, 48 h, 60 h and 72 h was counted, respectively, in MCF-7 (A), T47D (B), MDA-MB-231 (C) and Hs518bst (D). (TIF) [file pone.0046173.s003.tif]

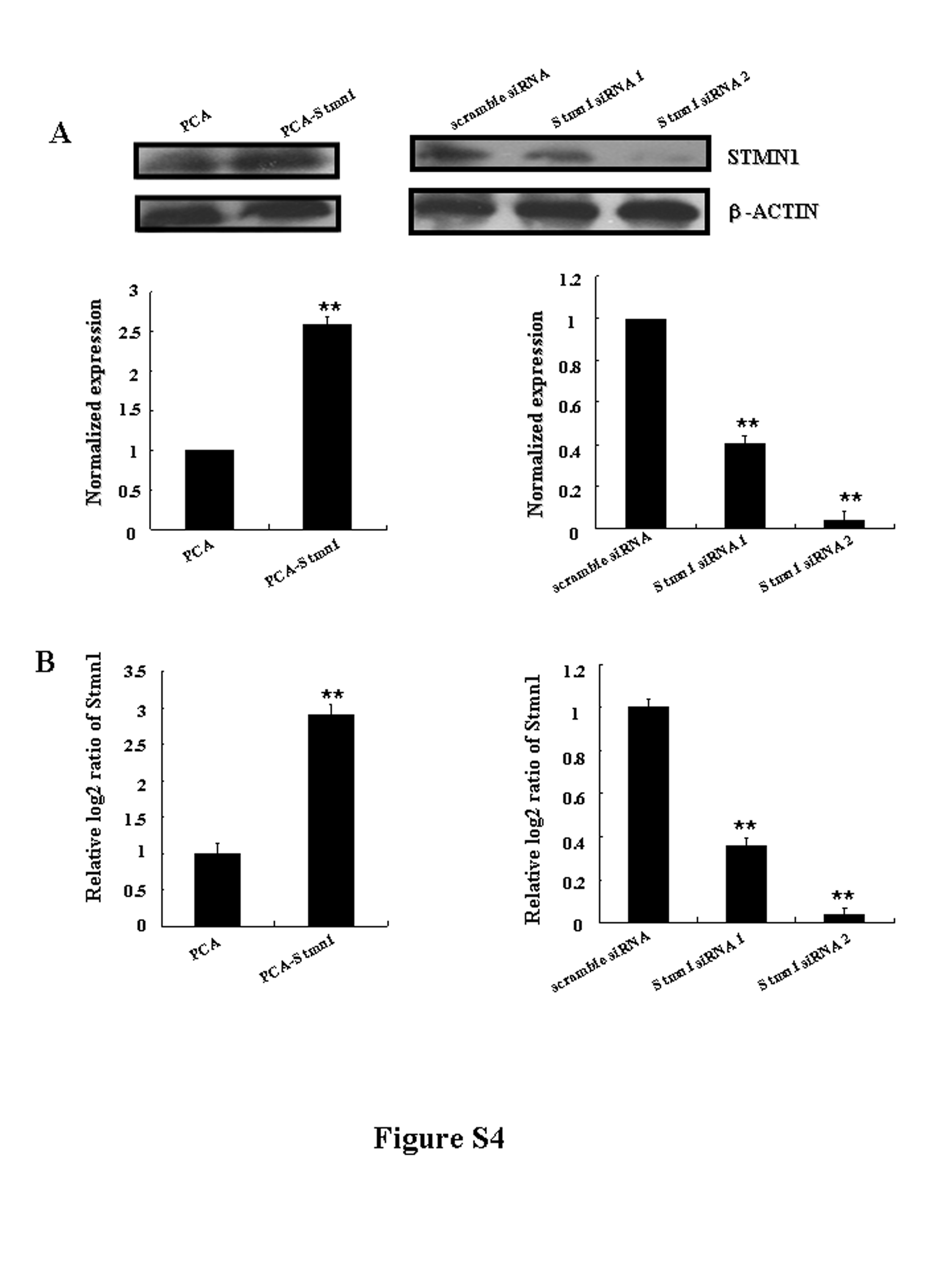

Supplement: Figure S4 — The effect of Stmn1 constructs on the expression level of Stmn1 . PCA or PCA-Stmn1 was transfected into MDA-MB-231 and scramble siRNA, Stmn1 siRNA1 or siRNA2 was transfected into Hs518bst. The STMN1 protein level was detected by western blot (A). The expression of β-ACTIN served as an internal control. The Stmn1 mRNA level was detected by RT-Real Time PCR (B). β-actin serves as an internal reference. **P<0.01. (TIF) [file pone.0046173.s004.tif]
